# Ionization Constants of Four Dinitrophenols in Water at 25 °C

**DOI:** 10.6028/jres.064A.034

**Published:** 1960-08-01

**Authors:** Robert A. Robinson, Marion Maclean Davis, Maya Paabo, Vincent E. Bower

## Abstract

Thermodynamic ionization constants of 2,3-, 2,5-, 3,4-, and 3,5-dinitrophenols in aqueous solution at 25° C have been determined by a spectrophotometric method. The respective values found, expressed as *p*K, are 4.95_9_, 5.21_0_, 5.42_2_, and 6.69_2_. *p*K has also been determined potentiometrically for 2,3- and 3,5-dinitrophenols; the respective values obtained are 4.98 and 6.66. The experimental *p*K values for all six dinitrophenols are lower than the calculated values based on *p*K data for phenol and the mononitrophenols.

Spectral absorption curves are presented for the ionized and unionized forms of the four dinitrophenols.

## 1. Introduction

“Concentration” ionization constants of the six dinitrophenols were determined by Holleman and Wilhelmy [1],^2^ and thermodynamic ionization constants have since been determined for all but the 2, 3- and 3,5-isomers. Only the 2,4- and 2,6-isomers are readily obtained commercially at the present time. The remaining isomers were synthesized in this laboratory for a study of the comparative acidic behavior of the six dinitrophenols in benzene.^3^ The same materials were used for the work reported in this paper; namely, the determination of thermodynamic *p*K values for the 2,3- and 3,5-isomers and redetermination of thermodynamic *p*K values for the 2,5- and 3,4-isomers.

## 2. Experimental Procedure

### 2.1. Materials

*3,5-Dinitrophenol* was made by converting 1,3,5- trinitrobenzene to 3,5-dinitroanisole [1, 2] and then demethylating [3]. The product was recrystallized from water as the dihydrate; this was dehydrated overnight in a vacuum oven, then recrystallized from benzene-cyclohexane and dried at 90° for one hour (mp, 124.3 to 125.0° C).

*2,3-, 2,5*-, and *3,4-Dinitrophenols* were prepared simultaneously by nitrating *m*-nitrophenol and then were separated by fractional crystallizations [1, 4]. Finally, each compound was recrystallized twice from the solvent indicated: 2,3-Dinitrophenol (from water), mp 146.5 to 147.0° C; 2,5-dinitrophenol (from 95% ethanol), mp 105.8 to 106.2° C; 3,4- dinitrophenol (from benzene), mp 135.1 to 135.5° C.

Potentiometric weight titrations for 2,3-dinitrophenol and for 3,5-dinitrophenol indicated a purity of not less than 99.8 percent.

### 2.2. Determination of *pK* values

The *spectrophotometric procedure* followed in determining the *p*K values was devised by Robinson and coworkers [5] in a research program one objective of which is to test the applicability of Hammett’s equation [6] to substituted phenols.

The *p*K of the dinitrophenol was calculated from the equation

$$pK=pH-\log[(D-D_{1})/(D_{2}-D)]-\log\gamma_{R^{-}}-\Delta pH.$$ (1)

The spectrophotometric data (see second term on right side of eq (1)) were obtained at 25.0±0.1° C with a Beckman Model DU quartz spectrophotometer, using optical absorption cells 1 cm in length. The symbols *D*_1_ *D*_2_, and *D* signify the spectral absorbances (optical densities) of solutions containing the same total molar concentration of dinitrophenol present as unionized molecules, phenolate ions, or mixtures of the two, respectively. In measuring the limiting spectral absorbances, the dinitrophenol was dissolved in aqueous hydrochloric acid of *p*H≈2 (for *D*_1_ values) or in aqueous sodium hydroxide of *p*H ≈ 12 (for *D*_2_ values). Measurements of *D* were made for at least three differently buffered solutions having known *p*H values which were close to the expected *p*K value of the dinitrophenol. The absorbances were measured at two or three wavelengths. Phosphate buffer mixtures [7] were used in determining the *p*K of 3,5-dinitrophenol, and succinate buffer mixtures [8] were used for the remaining dinitrophenols. The compositions and *p*H values of buffers used are given in tables 2 and 3.

The following modification of Davies’ equation [9] was used to calculate _γ*R*−_, the activity coefficient of the phenolate ion:^4^

$$-\log\;\gamma_{R^{-}}=A\surd I/(1+\surd I)-0.2\;I.$$ (2)

The final term in eq (1), Δ*p*H, is a correction applied because the addition of the phenol to the buffer mixtures causes some changes in the *p*H of the solution, a change which becomes more important as the buffer becomes more dilute [12]. This correction can be put in the form

$$\Delta pH\approx 0.4343[m_{3}/(m_{1}+m_{2})](K/K_{R})\times [{\left(K_{R}+h\right)}^{2}/(K+h)h],$$ (3)

where *m*_1_ and *m*_2_ are the molalities of the buffer salts, *m*_3_ is that of the phenol, *h* is the hydrogen ion concentration, *K_R_* is the ionization constant of the acid of the buffer, and *K* is the ionization constant of the phenol.

In the *potentiometric determination of p*K for 2,3- and 3,5-dinitrophenols, a 100-ml portion of 0.01-M aqueous solution of each compound was titrated at 25°±1° C with 0.1-M sodium hydroxide. The potential was measured between glass and saturated calomel electrodes [13].

Values of *p*K were computed from the equation

$$pK=pH-\log\{([B^{-}]+[H^{+}])/([HB]-[H^{+}])\}+\{(0.5115\surd I)/(1+1.5\surd I)\}.$$ (4)

The *p*K value obtained for 2,3-dinitrophenol was 4.98±0.005, and the *p*K value for 3,5-dinitrophenol was 6.66±0.001.

## 3. Results and Discussion

**Figure 1:**
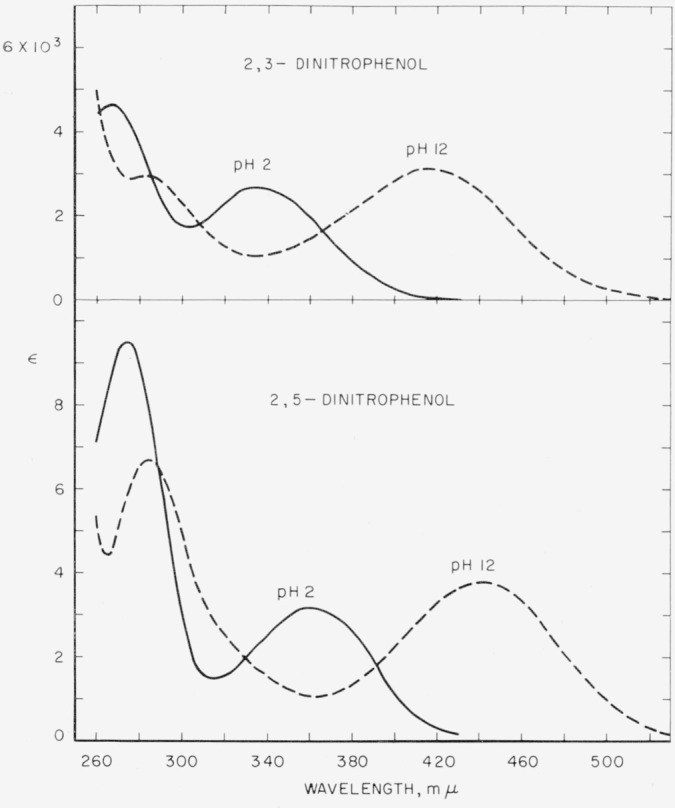
Absorption spectra of 2,3-dinitrophenol and 2,5- dinitrophenol in aqueous acid (p*H*≈2) and alkali (p*H*≈12).

**Figure 2:**
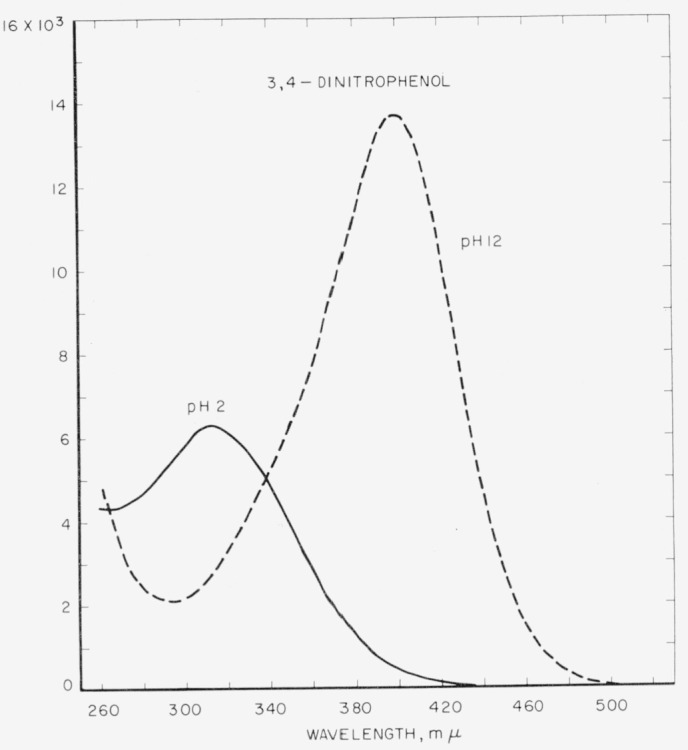
Absorption spectra of 3,4-dinitrophenol in aqueous acid (p*H*≈2) and alkali (p*H* ≈12).

**Figure 3:**
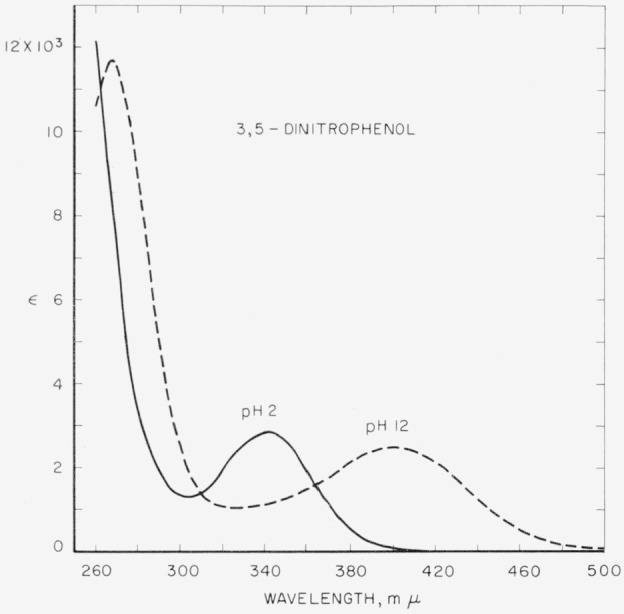
Absorption spectra of 3,5-dinitrophenol in aqueous acid (p*H*≈2) and alkali (p*H*≈12.)

Molar absorption curves for the dinitrophenols in aqueous acid and alkali are presented in figures 1 to 3. Optical constants are summarized in table 1.

Data and results for the ionization constants by the spectrophotometric procedure are summarized in tables 2 and 3.

In table 4, the *p*K values obtained in this work by the spectrophotometric method are compared with earlier experimental values. *p*K values for 2,4- and 2,6-dinitrophenols are included in table 4. It is evident that the thermodynamic ionization constants, expressed in *p*K units, agree well in general magnitude with the corresponding “concentration” ionization constants [1], but the thermodynamic values are from 0.01 to 0.14 *p*K unit higher. Our *p*K values for the 2,5- and 3,4-isomers are in excellent agreement with the results of Judson and Kilpatrick (table 4, footnote *g*).

Values of *p*K for all six dinitrophenols can be calculated from *p*K data for phenol itself and for the monosubstituted nitrophenols, using the assumption of additivity (see table 4). The experimental *p*K values are all lower than the calculated values, in agreement with an analogous conclusion of Holleman and Wilhelmy [1]. The disagreement is particularly marked in the case of dinitrophenols substituted in the 2-position.

## Figures and Tables

**Table 1:** Optical constants of dinitrophenols in aqueous acid and alkali

Compound	Absorption bands				Isosbestic points	
	*p*H=2		*p*H=12			

	λ_max_ m*μ*	*ε*_max_	λ_max_ m*μ*	*ε*_max_	λm*μ*	*ε*
						
2,3-Dinitrophenol	$\{\begin{array}{l} 265\\ 335\\ \ldots \ldots \ldots \\ \ldots \ldots \ldots \end{array}$	4620	282	2930	261	4500
		2700	416	a3130	285	2950
		……	……	……	308	1800
		……	……	……	366	1700
2,5-Dinitrophenol	$\{\begin{array}{c} 275\\ 360\\ \ldots \ldots \ldots \end{array}$	9500	285	6700	288	6550
		3150	440	a3770	330	2000
		……	……	……	392	1800
3,4-Dinitrophenol	$\{\begin{array}{c} 315\\ \ldots \ldots \ldots \end{array}$	6300	400	a13700	264	4300
		……	……	……	338	5000
3,5-Dinitrophenol	$\{\begin{array}{c} 343\\ \ldots \ldots \ldots \\ \ldots \ldots \ldots \end{array}$	2900	268	11700	262	11000
		……	400	a2480	310	1400
		……	……	……	364	1600

a G. Kortüm, Z. physik. Chem. **B42**, 39 (1939), reported values in good agreement with these results in the cases of 2,3-dinitrophenol (3160) and 3,5-dinitrophenol (2450), but not in the cases of 2,5-dinitrophenol (4170) or 3,4-dinitrophenol (5250). (See table on p. 64 of ref. cited.)

**Table 2:** Ionization constant of 3,5-dinitrophenol in water at 25° C

Ionic strength^a^	*p*H	−log *γ_R_*−	*D*^b^	$\log\frac{D-D_{1}}{D_{2}-D}$	*p*K	*p*K (corr.)
						
λ=400 m*μ* *D*_1_=0.037, *D*_2_=1.341						

0.2	6.772	0.118	0.830	0.191	6.699	6.693
.1	6.860	.103	.879	.260	6.703	6.691
.08	6.886	.097	.886	.271	6.712	6.696
.04	6.959	.077	.917	.318	6.718	6.686
.02	7.018	.059	.924	.328	6.749	6.687

λ=410 m*μ* *D*_1_=0.017, *D*_2_=1.298						

0.2	6.772	0.118	0.793	0.187	6.704	6.698
.1	6.860	.103	.839	.252	6.711	6.699
.08	6.886	.097	.851	.271	6.712	6.696
.04	6.959	.077	.883	.321	6.716	6.684
.02	7.018	.059	.889	.329	6.748	6.686

Avg						6.69_2_
*K*=2.03×10^−7^						

a The molar concentration of 3,5-dinitrophenol was 5.4×10^−4^. The buffer solutions contained equimolar KH_2_PO_4_ and Na_2_HPO_4_.

b The optical absorption cells were 1 cm in length. *D*_1_ and D_2_ symbolize, respectively, the spectral absorbance (optical density) of the unionized phenol and the phenol anion, and *D*, that of a solution containing a mixture of the two.

**Table 3:** Ionization constants of 2,3-, 2,5-, and 3,4-dinitrophenols in water at 25° C^a^

Buffer mixture No.^b^	2,3-Dinitrophenol 2.17×10^−4^ *M*				2,5-Dinitrophenol 1.13×10^−4^ *M*				3,4-Dinitrophenol 6.8×10^−5^ *M*			
	*D*	$\log\frac{D-D_{1}}{D_{2}-D}$	*p*K	*p*K (corr.)	*D*	$\log\frac{D-D_{1}}{D_{2}-D}$	*p*K	*p*K (corr.)	*D*	$\log\frac{D-D_{1}}{D_{2}-D}$	*p*K	*p*K (corr.)
			
	λ=410 m*μ* *D*_1_= 0.019, D_2_=0.671				λ=430 m*μ* *D*_1_=0.010, *D*_2_=0.414				λ=400 m*μ* *D*_1_=0.023, *D*_2_=0.930			
			
1	0.513	0.496	4.965	4.962	0.270	0.260	5.201	5.200	0.495	0.035	5.426	5.425
2	.524	.535	4.971	4.966	.276	.287	5.218	5.215	.519	.082	5.424	5.422
3	.534	.576	4.976	4.963	.286	.335	5.216	5.209	.548	.138	5.413	5.409
			
	λ=420 m*μ* *D*_1_=0.009, *D*_2_=0.676				λ=440 m*μ* *D*_1_=0.005, *D*_2_=0.426				λ=410 m*μ* *D*_1_=0.011, *D*_2_=0.866			
			
1	0.517	0.504	4.957	4.954	0.275	0.252	5.209	5.208	0.456	0.035	5.426	5.425
2	.527	.540	4.966	4.961	.284	.291	5.215	5.212	.474	.072	5.434	5.432
3	.536	.577	4.974	4.961	.291	.326	5.225	5.218	.500	.126	5.425	5.421
			
	λ=430 m*μ* *D*_1_=0.005, *D*_2_=0.632				Avg 5.21_0_ *K*= 6.17×10^−6^				Avg 5.42_2_ *K*=3.78×10^−6^			
	
1	0.483	0.506	4.955	4.952								
2	.493	.546	4.960	4.955								
3	.501	.580	4.971	4.958								
			
	Avg 4.95_9_ *K*= 1.10×10^−5^											

a Optical absorption cells 1 cm in length were used throughout. *D*_1_ and *D*_2_ signify, respectively, the spectral absorbance (optical density) of the unionized phenol and the phenol anion, and *D* symbolizes the absorbance of a solution containing both unionized and ionized phenol.

b Buffer mixtures Nos. 1, 2, and 3 were mixtures of *x*-molar sodium hydrogen succinate and *x*-molar disodium succinate, where *x*=0.05, 0.025, and 0.01, respectively. The *p*H values of these buffer mixtures were 5.343, 5.403, and 5.474, respectively.

**Table 4:** *p*K values of dinitrophenols in water at 25° C

Dinitrophenol	*p*K, calc a	*p*K, experimental				
		This work	Earlier work^b^			Δ*p*K^c^
						
2,3-	5.61	^*^4.96	4.89^d^	……	……	−0.65
2,4-	4.36	……	4.00^d^	4.09^e^	^*^4.11^f^	−.25
2,5-	5.61	^*^5.21	5.15^d^	5.22^g^	……	−.40
2,6-	4.42	……	3.57^d^	^*^3.71 h, i	……	−.71
3,4-	5.55	^*^5.42	5.37^d^	5.42^g^	……	−.13
3,5-	6.80	^*^6.69	6.68^d^	……	……	−.11

a Based on the following *p*K values: Phenol, 9.998; *o*-nitrophenol, 7.210; *m*-nitrophenol, 8.399; *p*-nitrophenol, 7.149. See R. A. Robinson and A. I. Biggs, Trans. Faraday Soc. **51**, 901 (1955); A. I. Biggs, Trans. Faraday Soc. **52**, 35 (1956).

b *p*K values determined at other temperatures are in general harmony with the values cited in this table. For example, L. Michaelis and A. Gyemant, Biochem. Z. **109**, 165 (1920); L. Michaelis and R. Krüger, Biochem. Z. **119**, 307 (1921); R. Riccardi and P. Franzosini, Boll. sci. fac. chim. ind., Bologna **15**, 25 (1957).

c Δ*p*K=^*^pK_exptl._*−p*K_calc._

d A. F. Holleman and G. Wilhelmy, Rec. trav. chim. **21**, 432 (1902). (R. Bader, Z. physik. Chem. **6**, 289 (1890), determined ionization constants for all of the dinitrophenols except 3,5-dinitrophenol, but only very approximately.)

e H. von Halban and G. Kortüm, Z. physik. Chem. **A170**, 351 (1934); see also W. D. Bale and C. B. Monk, Trans. Faraday Soc. **53**, 450 (1957).

f R. G. Bates and G. Schwarzenbach, Helv. chim. acta **37**, 1069 (1954).

g C. M. Judson and M. Kilpatrick, J. Am. Chem. Soc. **71**, 3110 (1949).

h G. Kortüm and H. Wilski, Z. physik. Chem. **2**, 256 (1954); earlier unpublished work of M. Kortüm-Seiler yielding the same result by a different method is cited.

i J. F. J. Dippy, S. R. C. Hughes, and J. W. Laxton, J. Chem. Soc. (London) 1**956**, 2995.

## Footnotes

2 Figures in brackets indicate the literature references at the end of this paper.

3 M. M. Davis and M. Paabo, work in progress.

4 The modification consists of substituting 0.2 *I* for 0.1 *I* [5]. *I* symbolizes the ionic strength. The value for the parameter *A* in the Debye-Hückel-Onsager equation has been recomputed because a more accurate value for the dielectric constant of water is available. The value of *A* adopted earlier [10] was 0.509; the new value is 0.5115 [11].
